# Non-canonical cell death in neurodegeneration: emerging mechanisms and therapeutic frontiers

**DOI:** 10.1007/s10495-026-02260-y

**Published:** 2026-02-16

**Authors:** Nilufer Ercin, Nail Besli, Merve Beker, Ulkan Celik

**Affiliations:** 1 Department of Medical Biology, Hamidiye School of Medicine, University of Health Sciences, Istanbul, Turkey; 2 Department of Medical Biology, Hamidiye International School of Medicine, University of Health Sciences, Istanbul, Turkey; 3 Department of Medical Biology, Institute of Health Sciences, University of Health Sciences, Istanbul, Turkey

**Keywords:** Cell death, Ferroptosis, Necroptosis, Neurodegenerative diseases, Parthanatos, Pyroptosis

## Abstract

Neurodegenerative diseases, specifically Alzheimer’s disease (AD), Parkinson’s disease (PD), Huntington’s disease (HD), and Amyotrophic Lateral Sclerosis (ALS) are defined by progressively increased neuronal loss that lacks curative therapies. Increasing evidence supports that non-canonical regulated cell death pathways including ferroptosis, necroptosis, pyroptosis, and parthanatos, are implicated in pathological mechanisms of neuroinflammation, and oxidative stress, and mitochondrial dysfunction, likely impacting neurodegenerative pathologies. In this review, we summarize the existing literature on the molecular pathways and potential pathogenic implications of these cell death pathways in neurodegenerative diseases, highlighting their upstream triggers, regulatory proteins, and downstream effectors. We also briefly describe representative pharmacological agents, including ferrostatin-1, necrostatin-1, MCC950 and PARP-inhibitors, that have shown neuroprotective effects in experimental studies. Experimental studies provide valuable information, but translation to clinical treatments presents barriers including overlapping regulated cell death mechanisms, constraints of bloodbrain barrier penetrance and concern for safety. Future development may come through concepts such as biomarker-based patient stratification strategies, multivalent interventions, and improved translational models. Identifying these new regulated cell death pathways may eventually provide new avenues to slow the progression of neurodegeneration and develop more targeted therapies.

## Introduction

Neurodegenerative disorders including Alzheimer’s disease (AD), Parkinson’s disease (PD), Huntington’s disease (HD), and Amyotrophic Lateral Sclerosis (ALS) are rising sharply with global ageing. By 2050, dementia alone is projected to impose a global cost of over US $9 trillion [1]. Despite improved insights into pathogenesis, available treatments remain only modestly effective [2, 3].

Canonical cell death apoptosis and regulated necrosis has long framed neurodegeneration research. Apoptosis engages intrinsic/extrinsic routes to activate executioner caspases and typically ends with immunologically quiet efferocytosis [4, 5]. Regulated necrosis, once deemed passive, includes programmed forms such as necroptosis driven by RIPK1/RIPK3/MLKL, linking death to inflammatory signaling [6]. By contrast, non-canonical pathways ferroptosis, necroptosis, pyroptosis, parthanatos carry distinct biochemical/evolutionary signatures and, unlike apoptosis, frequently release DAMPs/alarmins that amplify neuroinflammation [7–10]. Recent multi-omic analyses reveal dense crosstalk between canonical and non-canonical regulated cell death, with shared intermediates, reciprocal checkpoints, and hierarchical decision nodes. Ferroptosis-peroxisome coupling and PANoptosome assembly exemplify coordination among apoptosis, pyroptosis, and necroptosis [11, 12]. Accordingly, perturbing one pathway can reverberate across the broader death network, arguing for therapies that anticipate these interdependencies. Pathway engagement is also stage-dependent apoptosis may prevail early, while non-canonical programs increasingly dominate chronic/advanced disease supporting precision, time-resolved interventions [13].

Beyond canonical paradigms, recent work has increasingly implicated four regulated, non-apoptotic cell-death programmes in driving neuronal loss and neuroinflammation. These include ferroptosis, characterized by iron-driven lipid peroxidation [14–16], pyroptosis, mediated through Gasdermin-D-dependent inflammasome activation [17], necroptosis, executed via RIPK1-RIPK3-MLKL signalling [18], and parthanatos, initiated by PARP-1 overactivation [19]. Recognition of these pathways has opened new translational avenues, positioning them as tractable therapeutic targets to alleviate neuronal damage and improve patient outcome [20].

This review surveys recent therapeutic advances that modulate these non-canonical death programmes in neurodegenerative disease. We first outline their core machinery and disease phenotypes, then synthesize convergence and crosstalk, next discuss pathway-proximal pharmacodynamic anchors and delivery constraints, and finally propose an integrative framework that maps pathway nodes to cell-type programmes and candidate biomarkers to guide clinical translation.

**Fig. 1:**
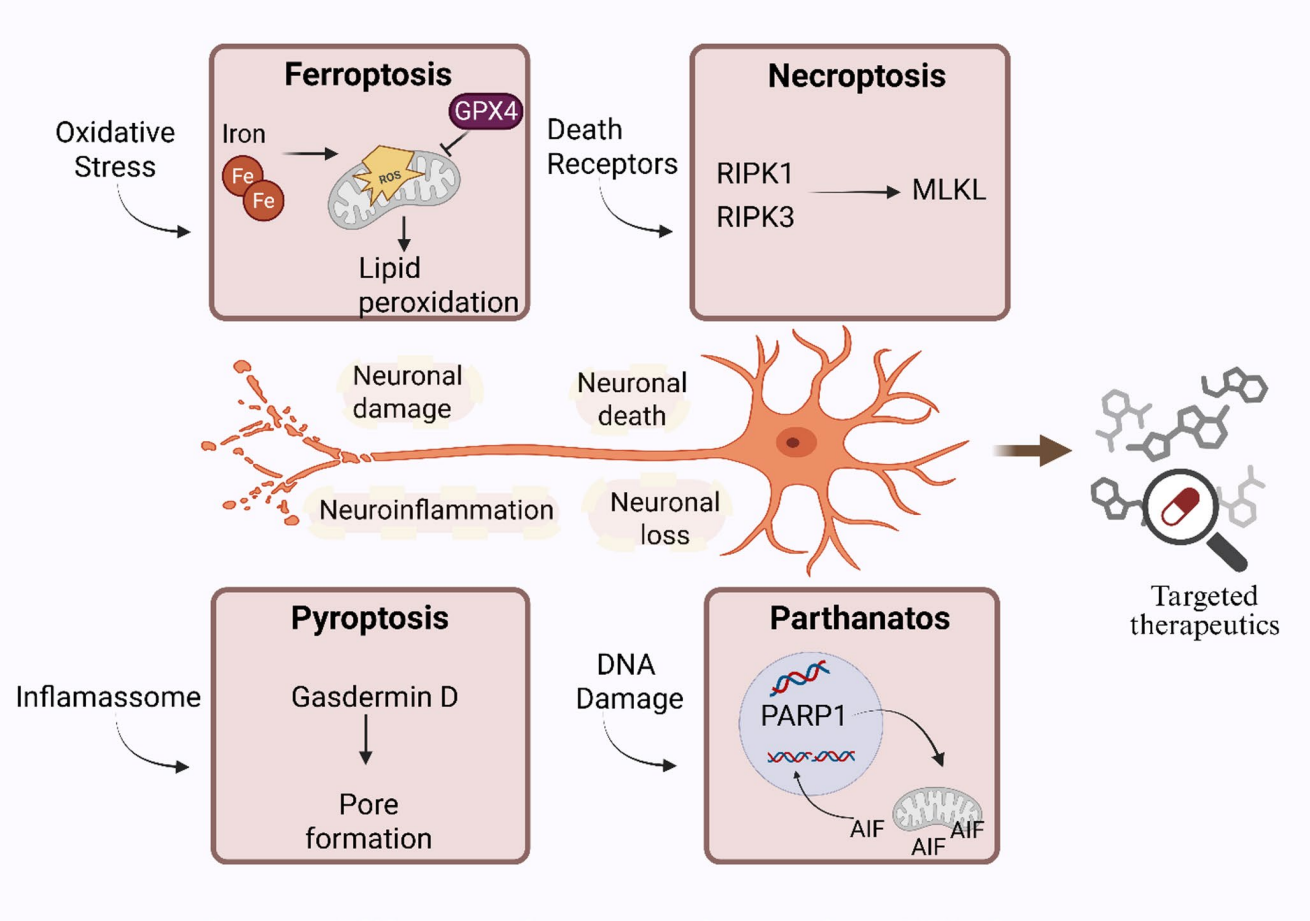
Overview of molecular mechanisms involved in non-canonical cell death pathways in neurons. This schematic illustrates the core signaling cascades and key effectors that define ferroptosis, necroptosis, pyroptosis, and parthanatos. Created in https://BioRender.com/5ujmmks

## Involvement of non-canonical cell death mechanisms in neurodegenerative disease

**Fig. 2:**
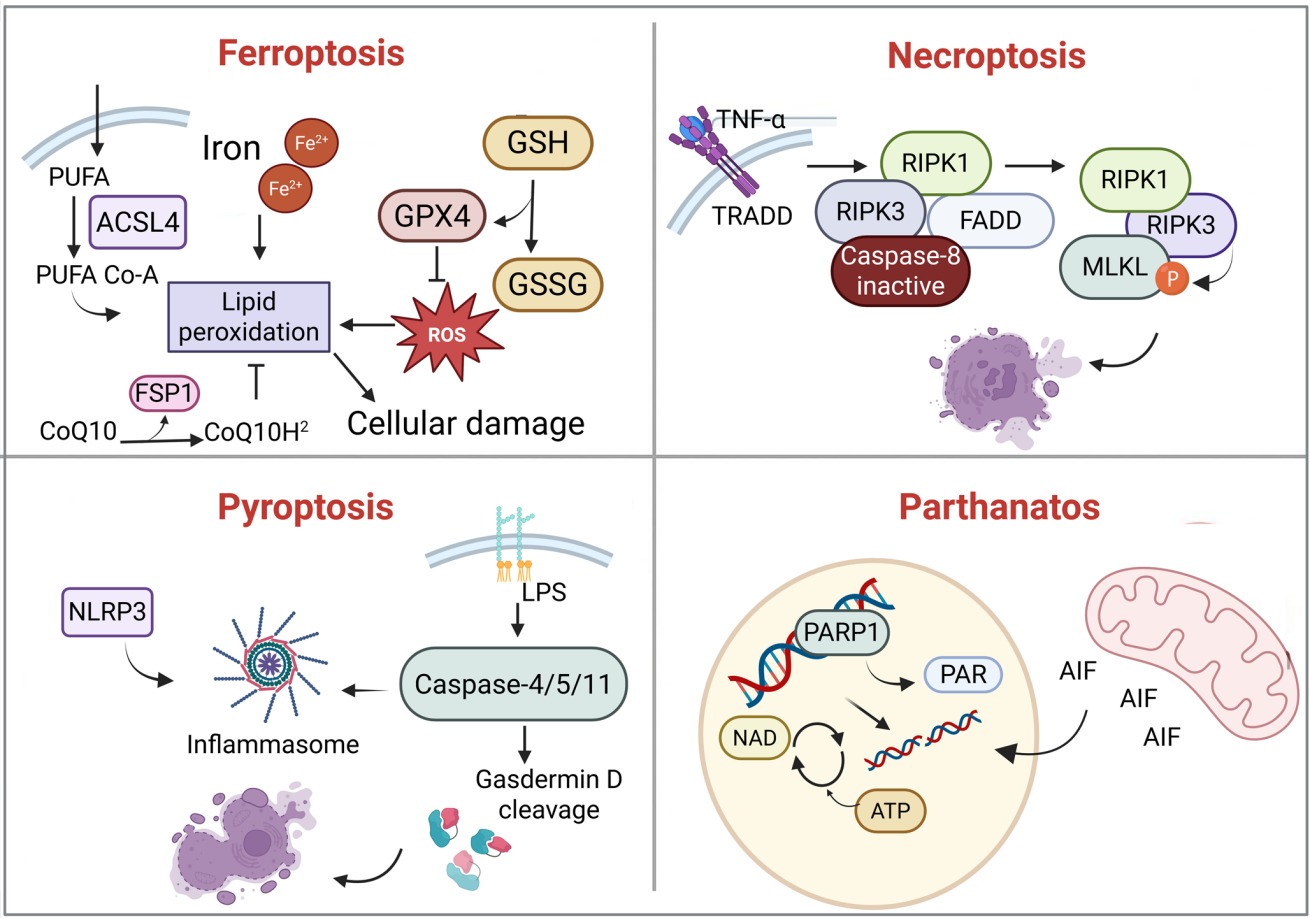
Interplay of non-canonical regulated cell death mechanisms in the pathology of neurodegenerative diseases. This figure depicts how ferroptosis, necroptosis, pyroptosis, and parthanatos contribute to disease progression in AD, PD, HD, and ALS. Pathway-specific triggers and cellular outcomes such as neuroinflammation, oxidative damage, and metabolic collapse are mapped across disease types. The diagram highlights the overlapping cellular stress responses and potential intervention points for therapeutic modulation of these pathways. Created in BioRender. https://BioRender.com/pzbivta

Growing evidence identifies the non-canonical death programmes as compelling therapeutic targets for neurodegenerative disorders [21–24]. Because these pathways converge on oxidative stress, neuroinflammation and mitochondrial dysfunction, intervening in them offers a fresh angle on the multifactorial nature of diseases such as AD, PD, HD, and ALS [25]. Studies have mapped druggable nodes within each pathway and shown that their inhibition confers marked neuroprotection in pre-clinical models [26–29] (Fig. 1). At the same time, integrative work is clarifying where lipid peroxidation and inflammatory signalling intersect, creating new opportunities for drug design and biomarker discovery [30]. An integrated schematic of these pathways and their roles in neuronal damage is illustrated in Fig. 2. In parallel, their key mechanistic features and distinguishing biomarkers are summarized in Table 1.

### **The core mechanisms of ferroptosis and its implications in neurodegeneration**

First described in 2012, ferroptosis is a regulated cell-death programme distinguished by iron-dependent lipid peroxidation rather than the caspase cascades typical of apoptosis or the membrane rupture seen in necrosis [31]. Its execution hinges on the interplay of iron handling, lipid metabolism and antioxidant defences [32]. Oxidation of membrane polyunsaturated fatty acids (PUFAs) generates lipid peroxides that, if not detoxified, compromise membrane integrity and trigger cell death [33]. Iron chelators can block this process, underscoring the central role of iron homeostasis and suggesting therapeutic potential in modulating intracellular iron levels [34, 35]. Two key protective systems counterbalance ferroptotic stress. First, glutathione peroxidase-4 (GPX4) reduces lipid hydroperoxides to non-toxic alcohols, a reaction that relies on glutathione (GSH). Second, the cystine/glutamate antiporter system Xc^−^ sustains GSH synthesis by importing cystine, its inhibition sensitises cells to ferroptosis [36–38]. Transcriptional activation of Nrf2 (NFE2L2) further bolsters antioxidant capacity by up-regulating detoxifying enzymes [36].

Ferroptosis is tightly coupled to cellular iron handling and oxidative stress, positioning it as a central mediator of neurodegenerative pathology [16]. Dysregulated iron homeostasis drives Fenton chemistry and PUFA lipid peroxidation, producing lethal lipid peroxide accumulation and membrane failure [16, 39, 40]. For disease-specific mechanistic summaries in AD, PD, ALS, and HD, see Sect. 3. The resulting redox imbalance heightens neuronal vulnerability and contributes to progressive loss in disorders such as AD and PD [41–43] This profile also anticipates the inter-pathway interactions detailed in Sect. 5, particularly where oxidative stress and mitochondrial dysfunction signaling converge.

**Table 1:** Comparison of mechanisms, biomarkers, and pharmacological agents

	Ferroptosis	Necroptosis	Pyroptosis	Parthanatos
Definition	Iron-dependent, lipid peroxidation-driven cell death	Caspase-independent, inflammatory necrotic death	Inflammatory cell death mediated by inflammasomes	PARP1-dependent cell death triggered by DNA damage
Triggers	Iron overload, erastin, RSL3	TNF-α, TLRs, interferons	LPS, nigericin, microbial infection	ROS, DNA damage, alkylating agents
Key Regulators	GPX4, xCT/SLC7A11, ACSL4, Nrf2, Fe²⁺	RIPK1, RIPK3, MLKL, caspase-8, FADD	NLRP3, Caspase-1/4/511, GSDMD	PARP1, AIF, PARG
Potential Biomarkers	ACSL4, lipid ROS, MDA	p-MLKL, RIPK3	GSDMD-N, IL-1β, caspase-1/4/511	PAR, nuclear AIF
Energy Dependence	Moderate (depends on GSH and NADPH)	Yes (requires ATP)	Yes (inflammasome activation)	Yes (ATP and NAD⁺ depletion)
Morphological aspects and Membrane Disruption	Cellular swelling, mitochondrial shrinkage, loss of cristae structure, and increased mitochondrial membrane density, and lipid ROS-induced lipid bilayer damage [8]	Cellular swelling, intermediate levels of chromatin condensation, and MLKL-mediated pore formation [8]	Cellular swelling, intermediate levels of chromatin condensation, and gasdermin D pore formation [8]	Shrinkage of the cell, AIF-mediated mitochondrial dysfunction, condensed chromatin, extensive DNA fragmentation, and compromised membrane integrity [77]
Therapeutic Inhibitors	Ferrostatin-1, Liproxstatin-1	Necrostatin-1 (RIPK1 inhibitor)	MCC950 (NLRP3 inhibitor), VX-765 (Caspase inhibitor)	PARP inhibitors (Olaparib, Veliparib)
Nomenclature Introduced	2012 [7]	2005 [7]	2001 [7]	2009 [7]

PARP-1: Poly(ADP-ribose) polymerase 1, RSL3: RAS-selective lethal 3, TNF-α: Tumor necrosis factor alpha, TLRs: Toll-like receptors, LPS: Lipopolysaccharide, ROS: Reactive oxygen species, GPX4: Glutathione peroxidase 4, xCT/SLC7A11: Cystine/glutamate antiporter, ACSL4: Acyl-CoA synthetase long-chain family member 4, Nrf2: Nuclear factor erythroid 2-related factor 2, RIPK1: Receptor-interacting protein kinase 1, RIPK3: Receptor-interacting protein kinase 3, MLKL: Mixed lineage kinase domain-like pseudokinase, FADD: Fas-associated protein with death domain, NLRP3: NOD-like receptor family pyrin domain containing 3, GSDMD: Gasdermin D, AIF: Apoptosis-inducing factor, PARG: Poly(ADP-ribose) glycohydrolase, MDA: Malondialdehyde, p-MLKL: phosphorylated-Mixed lineage kinase domain-like pseudokinase, GSDMD-N: N-terminal fragment of Gasdermin D, IL-1β: Interleukin-1 beta, PAR: Poly(ADP-ribose), GSH: Glutathione, NADPH: Nicotinamide adenine dinucleotide phosphate, ATP: Adenosine triphosphate, NAD⁺: Nicotinamide adenine dinucleotide, PARP: Poly(ADP-ribose) polymerase.

### The core mechanisms of necroptosis and its implications in neurodegeneration

Necroptosis is a caspase-independent, pro-inflammatory cell-death programme that becomes prominent when apoptotic signalling is blocked [44]. The loss of plasma membrane integrity and the induction of inflammatory responses link this process particularly to neurodegenerative diseases, infections, and inflammatory disorders. The RIPK1-RIPK3-MLKL axis constitutes the core signaling pathway of necroptosis [18]. Necroptosis is triggered by the activation of death receptors through the binding of death ligands such as TNF-α to receptors like TNFR1. Following this binding, Complex I is formed, which includes proteins such as RIPK1, TRADD, TRAF2, and cIAP1/2 [45]. This complex initially transmits cell survival signals via NF-κB [46]. Upon deubiquitination of RIP1 by CYLD, caspase-8, FADD, RIP1, and RIP3 assemble to form complex IIa. Within this complex, caspase-8 remains active and suppresses the necroptotic activity of RIP1 and RIP3, leading to apoptosis. However, when caspase-8 is inhibited or silenced, RIP1, RIP3, and MLKL associate to form the necrosome (complex IIb). In this context, the mutual direct or indirect phosphorylation between these components occurs [47]. Phosphorylated MLKL then oligomerises and inserts into the plasma membrane, disturbing ion homeostasis and causing swelling, membrane rupture and a burst of reactive oxygen species (ROS) [46] (Table 2).

**Table 2:** Biomarkers of non-canonical cell death pathways in neurodegenerative diseases

Death type	Biomarker type	Biomarkers	Role / mechanism
Ferroptosis	Lipid peroxidation products	MDA, 4-HNE	Indicators of oxidative stress and ferroptosis
Ferroptosis	Other lipid markers	F₂-isoprostanes, oxidized PE (PUFA-PE-OOH)	Products of ACSL4/lipoxygenase activity; ferroptosis-specific lipid peroxidation
Ferroptosis	Neuroimaging	QSM, T2 MRI	Detect iron deposits in brain regions
Ferroptosis	Iron transport proteins	Ferroportin	Regulates iron export; dysfunction causes accumulation
Ferroptosis	Antioxidant defenses	GPX4	Protects against lipid peroxides
Ferroptosis	Transcriptomic signatures	ACSL4, SAT1, ALOX12, TrioSig, FerrDb/KEGG panels	Reflect ferroptosis susceptibility and heterogeneity
Necroptosis	Phosphorylated proteins	pRIPK1, pRIPK3, pMLKL	Mark sequential stages of death signaling
Necroptosis	Necrosome complex	RIPK1-RIPK3-MLKL complex	Platform for necroptosis execution
Necroptosis (non-canonical)	Upstream mediators	TRIF, ZBP1	Bypass RIPK1, directly activate RIPK3-MLKL axis
Necroptosis (non-canonical)	Biomarker signature	pRIPK3, pMLKL, low/absent pRIPK1, TRIF/ZBP1	Distinguishes non-canonical necroptosis
Necroptosis	DAMPs	HMGB1	Released during necroptosis, measurable in fluids
Pyroptosis (non-canonical)	Effector proteins	Cleaved GSDMD (GSDMD-N)	Pore formation and membrane rupture
Pyroptosis (non-canonical)	Initiator caspases	Caspase-4/5 (human), Caspase-11 (mouse)	Direct sensing of cytosolic danger signals; cleave GSDMD
Pyroptosis	Inflammatory cytokines	IL-1β, IL-18 (CSF)	Released through GSDMD pores
Pyroptosis (alternative)	Effector proteins	Cleaved GSDME (GSDME-N)	Caspase-3 cleavage switches apoptosis-pyroptosis
Pyroptosis (alternative)	Initiator caspase	Caspase-8	Cleaves GSDMD under inflammatory stress
Parthanatos (non-canonical)	Nuclear events	AIF-MIF translocation	Nuclear co-translocation mediates large-scale DNA fragmentation
Parthanatos	PAR-protein complexes	PAR-α-synuclein	PAR interacts with α-syn aggregates
Parthanatos	Repair enzyme dysregulation	ARH3 deficiency, Ser-ADPr accumulation	Impaired reversal of ADP-ribosylation
Parthanatos	Metabolic markers	SARM1 activation, NAD⁺ depletion	Axonal degeneration through NAD⁺ collapse

MDA: Malondialdehyde, 4-HNE: 4-Hydroxynonenal, PE: Phosphatidylethanolamine, PUFA-PE-OOH: Polyunsaturated fatty acid–phosphatidylethanolamine hydroperoxide, ACSL4: Acyl-CoA synthetase long-chain family member 4, QSM: Quantitative susceptibility mapping, MRI: Magnetic resonance imaging, GPX4: Glutathione peroxidase 4, SAT1: Spermidine/spermine N1-acetyltransferase 1, ALOX12: Arachidonate 12-lipoxygenase, TrioSig: Ferroptosis-related gene signature (Trio signature), FerrDb: Ferroptosis Database, KEGG: Kyoto Encyclopedia of Genes and Genomes, pRIPK1: Phospho-receptor-interacting serine/threonine-protein kinase 1, pRIPK3: phosphorylated-Receptor-interacting protein kinase 3, pMLKL: phosphorylated-mixed lineage kinase domain-like pseudokinase, TRIF: TIR-domain-containing adapter-inducing interferon-β, ZBP1: Z-DNA-binding protein 1, HMGB1: High-mobility group box 1, DAMP: Damage-associated molecular pattern, GSDMD: Gasdermin D, GSDMD-N: N-terminal fragment of gasdermin D, IL-1β: Interleukin-1 beta, IL-18: Interleukin-18, CSF: Cerebrospinal fluid, GSDME: Gasdermin E, GSDME-N: N-terminal fragment of gasdermin E, AIF: Apoptosis-inducing factor, MIF: Macrophage migration inhibitory factor, PAR: Poly(ADP-ribose), ARH3: ADP-ribosylhydrolase 3, Ser-ADPr: Serine-ADP-ribosylation, SARM1: Sterile alpha and TIR motif-containing 1, NAD⁺: Nicotinamide adenine dinucleotide

Elevated membrane permeability and cytokine release place necroptosis at the intersection of neuroinflammation and neuronal loss [44]. Studies have guided treatments by reducing both neuronal loss and neuroinflammation.

In AD, elevated necroptotic signalling has been documented in patient brains and aligns with worsening cognition [48, 49]. Salvadores et al. emphasize in their study that amyloid- β peptides can trigger necroptotic pathways through microglial activation, a process that further spreads neuroinflammation and accelerates neuronal deterioration [48]. Experimental blockade of the RIPK1-RIPK3-MLKL axis in AD models limits neuronal death and helps maintain cognitive performance underscoring the pathway’s therapeutic appeal [50, 51]. Comparable observations have emerged in PD and vascular dementia: dopaminergic neurons and glia display necroptotic markers, and pathway inhibition affords measurable neuroprotection [52, 53]. The interplay between necroptosis and neuroinflammation is particularly critical; necroptosis in microglia promotes a chronic inflammatory phenotype [44, 54]. Necroptosis also intersects with oxidative and other cellular stress responses [55]. This suggests that multifaceted treatment strategies targeting necroptosis and associated inflammatory and oxidative cascades may be necessary for optimal benefit [56, 57].

These insights have sparked interest in small-molecule inhibitors of RIPK1, RIPK3 and MLKL, several of which confer neuroprotection in pre-clinical models and are advancing toward clinical evaluation [58] (Table 3). Beyond therapy, necroptotic markers detected in biofluids or imaging may aid prognosis and treatment monitoring [55, 59]. A fuller understanding of how necroptosis interfaces with oxidative and inflammatory stress should therefore inform both biomarker development and multipronged therapeutic strategies for neurodegenerative disease. This profile predicts inter-pathway interactions detailed in Sect. 5, particularly where oxidative stress signaling converges.

### The core mechanisms of pyroptosis and its implications in neurodegeneration

Pyroptosis is an inflammatory form of programmed cell death. The term combines *pyro* (“fire,” reflecting its pro-inflammatory nature) and *ptosis* (“falling,” as with other programmed deaths) [28]. It is typically initiated by innate immune stimuli that activate inflammasomes most prominently members of the NOD-like receptor (NLR) family and is associated with the rapid release of IL-1β and IL-18. Morphologically, cells often swell, the plasma membrane ruptures, and intracellular contents spill into the extracellular space, which can amplify local inflammation [60, 61]. Two main routes have been described. In the canonical pathway, sensors such as NLRP3 assemble an inflammasome that activates caspase-1. Caspase-1 cleaves gasdermin D (GSDMD) to liberate its N-terminal fragment (GSDMD-N), which oligomerizes in the plasma membrane to form pores and thereby drives pyroptotic death [62]. In the non-canonical pathway, human caspase-4/5 (and murine caspase-11) are directly engaged by intracellular lipopolysaccharide; molecules such as oxPAPC may compete with LPS and blunt this activation. These caspases also cleave GSDMD to generate pore-forming GSDMD-N. While they do not directly mature IL-1β or IL-18, the subsequent ionic fluxes (particularly K⁺ efflux) could permissively activate NLRP3 and lead to cytokine release [28].

Multiple factors appear to modulate pyroptosis. ROS and other stress signals can promote inflammasome priming or activation [63, 64], while NF-κB signaling frequently serves as a transcriptional “priming” step for pro-IL-1β and inflammasome components. Pathogens, toxins, and cytokine milieus further tune the response, suggesting substantial context dependence across tissues and disease states [65–67].

In the nervous system, pyroptosis seems to play a dual role. As part of host defense it may help contain infection; however, dysregulated or chronic activation can contribute to sterile inflammation and tissue injury [67–69]. Emerging literature links inflammasome activity and pyroptotic signaling to neurodegenerative settings for example, amyloid-β or tau in AD, dopaminergic vulnerability in PD, and related mechanisms in HD and multiple sclerosis [70–74]. In such contexts, sustained production of inflammatory mediators may create conditions that favor synaptic dysfunction, neuronal loss, and circuit impairment [75].

These insights have encouraged exploration of pharmacological modulators of inflammasome pyroptosis signaling. Inhibitors that target NLRP3, (e.g., MCC950, OLT1177/dapansutrile) have shown promise in experimental model systems and continue to be evaluated. Representative compounds and their reported effects are summarized in Table 3. This profile also predicts intra-pathway interactions, specifically where inflammatory signaling converges, which are detailed in Sect. 5.

### The core mechanisms of parthanatos and its implications in neurodegeneration

Parthanatos, derived from “poly(ADP-ribose) (PAR)” and the Greek word “Thanatos” (death), refers to a form of cell death caused by the accumulation of PAR polymers [76]. Parthanatos can be induced by various stressors, including ROS, DNA-damaging agents, nitric oxide (NO)-derived peroxynitrite, DNA alkylating compounds such as N-methyl-N’-nitro-N-nitrosoguanidine (MNNG), ultraviolet radiation, and ionizing irradiation [20]. Calcium overload triggered by glutamate receptor activation, along with ROS produced under oxidative stress, leads to both single- and double-stranded DNA breaks. These lesions result in the excessive activation of Poly(ADP-ribose) polymerase 1 (PARP-1). Poly(ADP-ribose) glycohydrolase (PARG), the main enzyme responsible for degrading poly(ADP-ribose) (PAR) chains, cleaves and releases small PAR oligomers from PARP1 and associated proteins. These PAR fragments subsequently migrate to the cytoplasm, where they interact with mitochondria, promoting the release of a cleaved form of apoptosis-inducing factor (AIF) from mitochondrial membranes. AIF then translocates to the nucleus partly guided by its nuclear localization sequence where it facilitates large-scale DNA fragmentation by activating endonucleases. A downstream bioenergetic crisis often accompanies this pathway, with NAD⁺ depletion, ATP loss, and broader metabolic impairment frequently described as hallmarks [77]. Neurons may be particularly vulnerable given their high energetic demand and limited regenerative capacity, which could help explain why parthanatos-like signatures are discussed in neurodegenerative contexts [78]. In AD, for instance, amyloid-β accumulation has been associated with oxidative stress, heightened PARP-1 activity, mitochondrial dysfunction, and indices of NAD⁺/ATP depletion findings that together suggest a potential parthanatos component and motivate interest in targeting PARP-1 [77, 79].

Experimental studies increasingly indicate that attenuating this pathway can be neuroprotective in vitro and in vivo models, although the magnitude and generalizability of benefit likely depend on context [80, 81]. Persistent PARP-1 activation under chronic oxidative stress and cumulative DNA damage has been linked to greater neuronal susceptibility, and dysregulated PARP-1 activity is sometimes correlated with disease severity, raising the possibility that it acts both as a marker and a mediator. These observations have encouraged exploration of PARP inhibitors in neurological settings [81–83]. Timing, dosing, and brain penetration appear critical variables, and several agents including olaparib and nicotinamide have been evaluated for effects consistent with dampening parthanatos-related injury (see Table 3 for examples in AD and PD models) [81, 84].

Looking ahead, closing the translational gap will likely require finer mapping of cell-type specific vulnerabilities, clarification of context-dependent triggers, and a better understanding of how parthanatos intersects with other regulated death programs such as ferroptosis and necroptosis. Parallel advances in more selective, CNS-penetrant PARP inhibitors and in biomarker strategies that enable real-time pathway monitoring could improve therapeutic precision. If successful, parthanatos modulation may eventually align with multi-target strategies aimed at slowing progressive neuronal loss.

## Disease focused summary of non-canonical cell death pathways in major neurodegenerative disorders

In AD, PD, ALS, and HD, non-canonical cell death converges on shared modules driven by iron-dependent lipid peroxidation in ferroptosis, RIPK1-RIPK3-MLKL signaling in necroptosis, NLRP3-GSDMD activation in pyroptosis, and PARP1-PAR-AIF following oxidative/DNA damage in parthanatos. Disease specific accents include Aβ-iron coupling in AD, α-synuclein lipid/iron interactions in PD, GPX4 dependency with RIPK1 regulated glial pathology in ALS, and striatal iron lipoxygenase programs in HD. These nodes frequently cross-regulate, so targeting one arm can shift others. Concise, disease-resolved summaries are provided in Sect. 3.1–3.4, with pathway crosstalk detailed in Sect. 5.

### Alzheimer’s disease

Ferroptosis is mechanistically driven by cortical/hippocampal iron dyshomeostasis, PUFA rich membrane vulnerability, and repression of the SLC7A11/GSH/GPX4 axis, which converge to heighten neuronal lipid-peroxidation stress and are further exacerbated by microglial neuron crosstalk. In AD models, iron modulating strategies, radical trapping antioxidants, and Nrf2/GPX4 supportive interventions blunt ferroptotic lipid ROS and mitigate synaptic injury [85]. Necroptosis proceeds via death-receptor/TLR signaling that activates RIPK1, recruits RIPK3, and phosphorylates MLKL to permeabilize membranes; elevations of phospho-RIPK1-RIPK3-MLKL in AD tissue and pathway blockade reducing neuronal/synaptic injury in models support causality [57]. Pyroptosis is a major driver of neuroinflammation in AD. The process is orchestrated by inflammasomes particularly NLRP1 and NLRP3 and is triggered by stressors such as amyloid-β aggregates. Aβ promotes NLRP3 assembly and caspase activation, leading to gasdermin D cleavage and formation of N-terminal pores in the plasma membrane. The ensuing ion flux and membrane rupture release IL-1β and IL-18, amplifying local inflammation and contributing to neurodegeneration [73, 86]. The initiation of parthanatos occurs when neurons undergo oxidative stress, often driven by the accumulation of toxic metabolites such as amyloid-β plaques a hallmark of AD [87]. These stressors cause DNA damage, leading to hyperactivation of PARP-1. Upon activation, PARP-1 synthesizes PAR polymers which serve as signals for DNA repair under physiological conditions but, when they accumulate excessively, act as triggers for programmed cell death [88, 89].

### Parkinson’s disease

Ferroptosis in PD stems from substantia nigra iron overload and PUFA-rich membrane vulnerability together with collapse of anti-ferroptotic defenses System Xc^−^-GSH-GPX4, FSP1/CoQ10 (and mitochondrial DHODH), and GCH1-BH4 and is exacerbated by DJ-1 dysfunction and reduced CoQ10/BH4. Iron accelerates α-synuclein aggregation and phosphorylation (via IRP/IRE control and kinase modulation), while α-synuclein reciprocally promotes Fe^3+^-Fe^2+^ reduction, lipid peroxidation, and mitochondrial injury through VDAC/mPTP interactions creating a ROS-driven feed-forward loop. Iron chelation, radical-trapping antioxidants, and Nrf2/GPX4-supportive strategies attenuate dopaminergic injury in models [90, 91]. Necroptosis proceeds via death-receptor/TLR signaling that activates RIPK1, recruits RIPK3, and phosphorylates MLKL to permeabilize membranes; elevated RIPK1-RIPK3-p-MLKL and pathway blockade reducing dopaminergic loss in experimental PD support causality [92]. Pyroptosis is fueled by α-synuclein aggregates that trigger NLRP3 assembly and caspase-1 activation in microglia, leading to GSDMD pore formation and IL-1β/IL-18 release; pharmacologic NLRP3 inhibition dampens inflammatory injury in PD models [93]. Parthanatos is initiated by oxidative/DNA-damage stress that hyperactivates PARP-1, driving PAR accumulation and AIF translocation; in PD models (MPP⁺/MPTP; α-syn PFF), PARP-1 or PAAN/MIF-axis inhibition limits neurodegeneration, indicating a druggable PARP1, AIF death program [94–96].

### Huntington’s disease

Ferroptosis in HD, striatal vulnerability converges on iron dysregulation and PUFA rich membrane stress with impaired antioxidant defenses; imaging and cohort work indicate basal ganglia iron accumulation from premanifest stages, while mechanistic studies identify ALOX5 as a key driver of mHTT-linked, ACSL4 independent ferroptosis, nominating lipoxygenases and GPX4/SLC7A11 support as actionable nodes [97–99]. Necroptosis, HD-like toxin models (3-nitropropionic acid) show robust activation of the RIPK1-RIPK3-MLKL axis in striatum, and pathway interference lessens behavioral and histologic injury supporting a contributory role of necroptosis in HD relevant contexts [100]. Pyroptosis, striatal pyroptotic signaling (NLRP3-caspase-GSDMD) is reported in R6/2 mice, with additional work highlighting NLRP3-centered neuroinflammation as a disease mechanism and candidate target in HD [101]. Parthanatos biomarkers PARP1-PAR-AIF axis appears dysregulated rather than uniformly upregulated in HD. Recent clinical biomarker studies show reduced CSF (cerebrospinal fluid) PAR in mutation carriers, suggesting altered PARylation dynamics despite persistent DNA damage stress, and keeping PARP1-directed strategies under active debate. Mechanistic reviews still place PARP1 hyperactivation and AIF translocation at the core of parthanatos, underscoring disease and context dependence for HD [102, 103].

### Amyotrophic lateral sclerosis

Ferroptosis in ALS is supported by motor-neuron lipid-peroxidation vulnerability with GPX4 dependence, GPX4 loss or knockdown drives ALS like paralysis, while GPX4 overexpression or anti-ferroptotic interventions suppress lipid ROS and preserve motor neurons in models [104, 105]. Necroptosis features engagement of the RIPK1-RIPK3-MLKL axis and a RIPK1 regulated inflammatory glial state. Genetic or pharmacologic RIPK1 blockade mitigates neuroinflammation and slows disease in SOD1^G93A^ mice, while human ALS tissues show elevated RIPK1 activity/signatures [106]. Pyroptosis related signals (inflammasome-NLRP3/GSDMD) have been detected in ALS spinal cord and glia; multiple studies suggest astrocytic involvement and microglial GSDMD activation, with NLRP3/GSDMD colocalization in ALS models and increased GSDMD in post-mortem tissue. However, GSDMD deletion in SOD1^G93A^ mice yields only modest survival effects and minimal changes in gliosis or motor-neuron loss, indicating that pyroptosis contributes in a context-dependent manner and that upstream inflammasome nodes or alternative gasdermins may represent more relevant therapeutic targets [107–109]. Reviews and experimental data in ALS highlight increased PARP1 activity/AIF signaling and DNA repair stress in motor neurons and glia, nominating the PARP1-PAR-AIF axis as a modifiable death program [89, 103, 110].

## Comprehensive biomarkers of non-canonical cell death pathways in neurodegeneration

Pathway-specific biomarkers in biofluids or tissue can enable earlier diagnosis, sharper disease monitoring, and mechanism-guided therapy in neurodegeneration. Integrating molecular, metabolic, and transcriptomic readouts provides a multidimensional view of regulated cell death. Recent discoveries highlight distinct biomarker signatures for each pathway, offering tools for early intervention and personalized treatment. Table 2 summarizes the major non-canonical cell death biomarkers in neurodegeneration and their mechanistic roles.

### Ferroptosis biomarkers

A broad spectrum of ferroptosis-related biomarkers has been identified, reflecting different mechanistic layers of this iron-dependent form of regulated cell death. As summarized in Table 1, these biomarkers span from classical lipid peroxidation products to molecular regulators.

Ferroptosis-related biomarkers provide valuable insight into the mechanisms of neurodegenerative diseases. Classic markers of lipid peroxidation, such as malondialdehyde (MDA) and 4-hydroxynonenal (4-HNE), are widely used indicators of oxidative stress and ferroptosis. Elevated MDA levels are linked to PD and AD, while 4-HNE accumulation is associated with synaptic and mitochondrial dysfunction, making both valuable markers for early diagnosis and disease monitoring [111–113]. Other lipid markers, such as F₂-isoprostanes well-established indicators of in vivo lipid peroxidation and oxidized phosphatidylethanolamine (PE) species, particularly PUFA-containing PE-OOH, are closely associated with ferroptosis due to their mechanistic link to iron-dependent lipid peroxidation. These compounds, produced through ACSL4 and lipoxygenase activity, highlight specific lipid peroxidation pathways that drive cell death and could serve as precise markers in clinical trials [114]. Neuroimaging tools such as quantitative susceptibility mapping (QSM) and T2 MRI show increased iron deposits in regions like the hippocampus, cortex, and substantia nigra in AD, PD, and HD. These findings match pathological data and support the idea that excess iron accelerates ferroptosis and neuronal loss [115–117]. From a molecular perspective, iron transporter proteins such as ferroportin are key factors regulating ferroptosis. Decreased ferroportin levels predict progression of AD, while ferroportin dysfunction leads to harmful iron accumulation in [118]. Antioxidant defenses also matter. GPX4 is a crucial enzyme that protects neurons from lipid peroxides. Loss of GPX4 causes rapid neurodegeneration [119]. Finally, transcriptomic studies have identified panels of ferroptosis-related genes (e.g., ACSL4, SAT1, ALOX12) that serve as diagnostic and prognostic markers in AD and ALS. These gene signatures reflect the molecular diversity of ferroptosis and may help in patient classification and therapy monitoring. Transcriptomic studies have identified ferroptosis-related gene signatures, including TrioSig and panels from FerrDb/KEGG, enriched in AD, while single-cell analyses show SAT1 and FTH1 upregulation in astrocytic ferroptosis [120, 121]. These gene signatures reflect the molecular diversity of ferroptosis and may help in patient classification and therapy monitoring.

### Necroptosis biomarkers

In necroptosis, pRIPK1, pRIPK3, and pMLKL, in particular, mark sequential steps of death signaling, and their phosphorylated status has been confirmed in AD, multiple sclerosis, and ALS brain tissues [18]. The necrosome complex, found in granulovacuolar inclusions, further highlights necroptosis in AD and HD [122]. Non-canonical necroptosis proceeds through alternative pathways that bypass RIPK1 but center on the RIPK3-MLKL axis. In particular, TLR3/4-TRIF-mediated signaling and the activation of ZBP1 (Z-DNA binding protein 1) directly trigger RIPK3, thereby initiating necroptotic cell death. Therefore, TRIF and ZBP1 expression profiles are suggested as specific indicators of non-canonical necroptosis [123]. TRIF and ZBP1 expression profiles have been proposed as specific indicators of non-canonical necroptosis. In viral infection models, upregulation of ZBP1 has been confirmed, while in autoimmune and neuroinflammatory diseases, tissue-level activation of the TLR3/4-TRIF-RIPK3 axis has been validated [44]. pRIPK3 and pMLKL are important biomarkers, while RIPK1 phosphorylation remains absent or at low levels, providing a distinguishing clue from the canonical pathway. Therefore, the combination of ‘pRIPK3 and pMLKL positivity, absence of pRIPK1, and upregulation of TRIF/ZBP1 constitutes a distinctive biomark [124]. HMGB1, a typical damage-associated molecular pattern (DAMP), provides a measurable fluid biomarker reflecting tissue damage and systemic inflammation [125].

Integrating these biomarker signatures into personalized medicine may enable the stratification of patients with neurodegenerative disorders in which non-canonical necroptosis predominates, thereby guiding prognosis and the development of targeted RIPK3/MLKL-based therapies.

### Pyroptosis biomarkers

Cleaved GSDMD (GSDMD-N) and activated caspase-4/5 in humans and caspase-11 in mice represent central indicators of neuronal pyroptosis triggered by cytosolic danger signals. While direct evidence from human AD or PD brain tissue is still emerging, foundational mechanistic work confirms that caspase-4/5/11 cleave GSDMD to GSDMD-N in non-canonical pyroptosis [126]. In AD, microglial caspase-4 is upregulated via DNA hypomethylation, linking this pathway to pathology [127, 128]. In PD, GSDMD contributes to dopaminergic neurodegeneration and glial activation [68]. Elevated cerebrospinal fluid levels of IL-1β and IL-18 provide fluid-based evidence of this pathway [129]. In addition, caspase-3 mediated cleavage of GSDME (GSDME-N) links apoptosis-to-pyroptosis switching to synaptic dysfunction in AD [130], while caspase-8 mediated GSDMD cleavage has been demonstrated as an alternative pyroptotic route under inflammatory stress [131]. Together, these biomarkers reflect distinct but converging pathways of non-canonical pyroptosis in the brain. Importantly, their integration into personalized medicine frameworks may enable stratification of patients according to dominant pyroptotic mechanisms, facilitate early diagnosis through cerebrospinal fluid or imaging-based assays, and support tailored interventions aimed at selectively blocking caspase-4/5, GSDMD, or GSDME activation. Such biomarker-guided strategies hold potential to refine prognosis and optimize targeted therapies in AD, PD and related neurodegenerative diseases.

### Parthanatos biomarkers

In neurodegenerative diseases, non-canonical branches of parthanatos define biomarker signatures with direct implications for personalized medicine. Nuclear AIF-MIF translocation, PAR-α-synuclein complexes, altered CSF PAR levels, ARH3 deficiency with serine-ADP-ribosylation, and SARM1-driven NAD⁺ depletion each represent distinct molecular readouts of parthanatos activity [76, 132–134]. Importantly, these biomarkers may be instrumental in patient stratification. Individuals showing AIF-MIF positivity may benefit from MIF or PARP1 inhibitors, high PAR or PAR-α-synuclein signatures may indicate suitability for PARP-targeted therapies, and NAD⁺/ARH3 imbalances may indicate SARM1 inhibition or NAD⁺ supplementation. Consequently, by linking biomarker profiles to treatment choices, it provides a framework for personalizing interventions, improving prognosis, and advancing precision medicine in diseases related to neurodegeneration.

## **Crosstalk between non-canonical cell death**

The non-canonical forms of programmed cell death ferroptosis, necroptosis, pyroptosis, and parthanatos exhibit distinct molecular mechanisms; however, an increasing number of studies suggest that these pathways may dynamically interact with one another. This crosstalk plays a significant role in the pathophysiology of various diseases, particularly neurodegenerative disorders [135, 136].

Ferroptosis and necroptosis interactions seem to coalesce at ROS and iron management. Ferroptosis induces iron-dependent lipid peroxidation and membrane damage, whereas necroptosis proceeds via RIPK1-RIPK3-MLKL signaling. Iron overload can increase ROS and sensitize cells to both processes, and lipid peroxidation products formed during ferroptosis may also prime cells for activation of the RIPK1-RIPK3-MLKL axis. Necroptotic damage may also release labile iron, which could feedback to promote ferroptotic sensitization [137, 138]. Pharmacological data (e.g., necrostatin-1 cross-effects) further illustrate this axis, although off-target actions complicate interpretation Notably, neuronal death after hemorrhagic stroke engages both ferroptosis and necroptosis, with inhibition of either pathway conferring more than 80% neuroprotection [139]. During early reperfusion after ischemic stroke, treatment with the ferroptosis inhibitor liproxstatin-1 suppressed necroptotic signaling, while the necroptosis inhibitor necrostatin-1 reduced ferroptosis-related protein alterations. Similarly, the iron chelator deferoxamine mesylate inhibited both processes. Together, these findings underscore a therapeutically exploitable interplay that may be addressed through combined strategies, such as pairing lipid peroxidation inhibitors (ferrostatin-1 or iron chelators) with RIPK1/RIPK3 blockade. Still, pharmacological specificity is essential, as RIPK3 expression varies across cell types and may influence necroptotic responses [137].

Links to pyroptosis have likewise been proposed. Inflammation and oxidative stress can accompany ferroptosis, and lipid peroxidation together with glutathione depletion may favor inflammasome activation and GSDMD-mediated pyroptosis [140]. In traumatic brain injury, PAR1 signaling emerges as a shared upstream node linking ferroptotic lipid-peroxidation/iron-redox stress with NLRP3 inflammasome mediated pyroptosis. PAR1 antagonism (SCH79797) concurrently reduces ferroptosis (via PPAR-γ/Nrf2-HO-1, preserving GPX4/SOD) and suppresses NLRP3-ASC-caspase-1 and downstream cytokines, highlighting PAR1 as a tractable lever to co-modulate both pathways [141].

Ferroptosis-related oxidative stress can also produce DNA damage, which may hyperactivate PARP1 and engage parthanatos [142, 143]. This DNA-damage link highlights how ferroptotic processes may spill over into additional death programs under conditions of severe redox imbalance.

Additional connections are noted between necroptosis and pyroptosis: activated MLKL disrupts the plasma membrane and promotes K⁺ efflux, a well-recognized permissive signal for NLRP3 inflammasome activation and subsequent pyroptotic responses [144]. This MLKL-K⁺-NLRP3 relay has been demonstrated across multiple systems and refined in recent structural/functional syntheses [145, 146]. In the pyroptosis arm, GSDMD pores drive rapid ionic dysregulation most notably K⁺ efflux, which further licenses NLRP3 activation; downstream, the membrane-rupture protein NINJ1 oligomerizes to execute terminal plasma-membrane rupture across lytic deaths (pyroptosis, necroptosis), thereby amplifying DAMP release and inflammation [147, 148]. Consistently, in an OGD cell model, the necroptosis inhibitor ZJU-37 (RIP1/RIP3 dual inhibitor) suppressed NLRP3 inflammasome activity [149].

Finally, ROS generated during necroptosis or pyroptosis can exacerbate DNA damage and PARP1 activation, linking these processes to parthanatos-like injury via NAD⁺/ATP depletion and AIF translocation [150, 151]. Supporting this overlap, inflammation-associated ROS in pyroptosis may also engage PARP1-dependent cell death [143, 151]. In a HD mouse model, PARP-1 inhibition was shown to reduce caspase-1 dependent pyroptosis, lowering NLRP3 expression and influencing neuronal morphology [152].

**Fig. 3:**
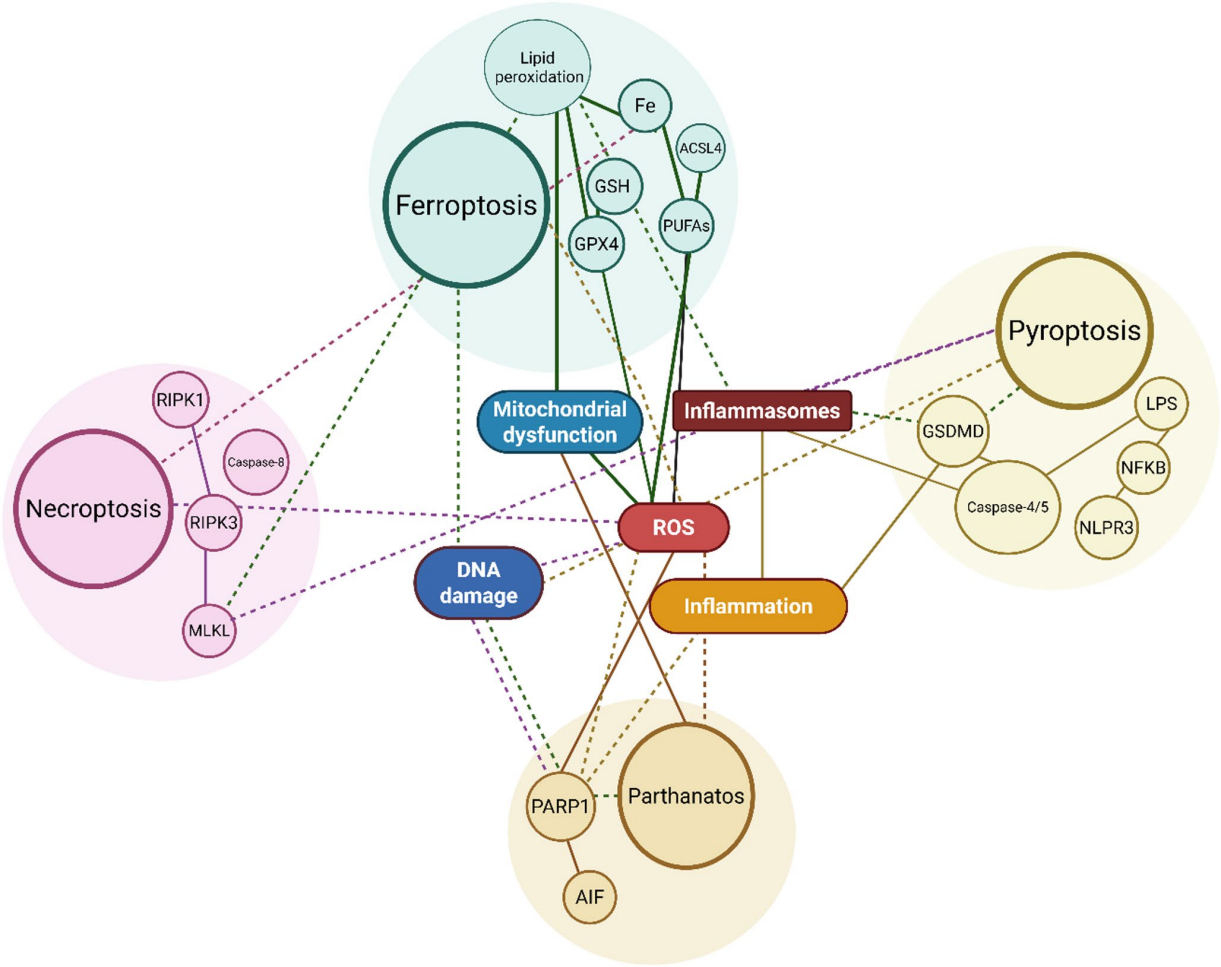
Interaction network among non-canonical cell death pathways. This schematic diagram illustrates the molecular interactions of ferroptosis, necroptosis, pyroptosis, and parthanatos, along with their connections to shared pathological processes. Solid lines represent direct molecular interactions and biochemical relationships. Dashed lines indicate indirect associations or the influence of one pathway on another. Created in https://BioRender.com/1bv771o

Taken together, these multi-directional interactions highlight therapeutically targetable nodes across death pathways. While each pathway offers potential intervention points, overlapping mechanisms also pose challenges, including pharmacological specificity and context-dependent signaling. Overall, regulated death programs do not function in isolation but rather form a dynamic network that shapes disease progression (Fig. 3). Understanding this crosstalk may open new therapeutic avenues, particularly in conditions where multiple cell death mechanisms are simultaneously activated.

## Emerging therapeutic strategies and pharmacological agents for non-canonical cell death mechanisms

Pharmacological suppression of ferroptosis has emerged as a potential strategy for neuroprotection, supported by preclinical studies that identify small-molecule inhibitors targeting key nodes in ferroptotic signaling [153, 154]. In neuronal cultures, such agents appear to curb lipid peroxidation and help preserve mitochondrial function, in part by limiting free-radical propagation and interrupting the self-amplifying loop of lipid damage; together, these effects may reduce ferroptosis-linked cell death [155–157]. These properties suggest possible therapeutic value in complex brain disorders where oxidative stress and inflammation frequently coexist.

The complex biochemistry underlying ferroptosis provides multiple potential targets for drug discovery, including enzymes and ion channels involved in iron transport and lipid oxidation. Research indicates that iron chelators and lipoxygenase inhibitors can significantly mitigate ferroptotic cascades by reducing free iron availability and limiting enzymatic lipid peroxidation [158]. Agents that support GPX4 activity or otherwise bolster antioxidant defenses have also been explored [158]. Because mitochondrial dysfunction features prominently in ferroptosis and often tracks with neurodegenerative pathology, it represents an attractive therapeutic focus [159]. Across models, mitochondrial injury coincides with elevated ROS, further fueling lipid peroxidation and death [160, 161]. Inhibitors that blunt ferroptosis have been reported to improve mitochondrial respiration and lower ROS generation, with accompanying signals of better energy homeostasis [162]. and associations with enhanced cell survival and reduced degenerative markers [163]. Thus, targeting mitochondrial impairment via ferroptosis-directed agents may help sustain neuronal viability.

Preclinical work also notes that ferroptosis antagonists can lower lipid-peroxidation readouts in neural tissues [153], while small molecules that restore glutathione reinforce endogenous brakes on ferroptotic progression [164]. These synergistic effects suggest that a combined approach of ferroptosis inhibitors and redox modulators may provide robust neuroprotection.

Pharmacological approaches aimed at necroptosis have shown encouraging signals in preclinical neurodegeneration models. Small molecules such as necrostatin-1 have been widely studied for interference with RIPK1 signaling, thereby limiting downstream RIPK3 and MLKL activation [11, 114]. Agents such as dabrafenib and ponatinib have been reported to reduce tau pathology and amyloid burden while preserving synaptic function in AD models, alongside attenuation of neuroinflammation and maintenance of neuronal integrity [51, 59]. Even so, concerns regarding target specificity and off-target liabilities persist, underscoring the need for further optimization of dosing and selectivity [50, 165].

Beyond direct cytoprotection, necroptosis inhibition may also temper inflammation, as membrane rupture can release damage-associated molecular patterns (DAMPs) that amplify neuroinflammatory cascades [50, 59, 166]. Consistent with this, RIPK1 inhibitors have been shown to reduce neuronal loss together with pro-inflammatory cytokine production in experimental systems, supporting combined strategies that address both cell-death signaling and inflammation [167, 168].

A substantial body of evidence indicates that modulation of pyroptotic pathways holds significant potential for mitigating neurodegenerative pathologies. Pharmacological inhibition of caspase activation, particularly targeting caspase-4/5/11, has been demonstrated to attenuate pyroptosis-associated neurotoxicity and ameliorate neuropathological features in various preclinical models [169, 170]. A number of more recent initiatives included both natural products and synthetic products, including alkaloids from plant producers that favor antioxidative/anti-inflammatory pathways that may still serve to indirectly inhibit pyroptotic signaling but also products that target inflammasome-related pathways and upstream players [170, 171].

The link between pyroptosis and neuroinflammation appears especially tight in glial circuits. Microglia and astrocytes are essential for CNS homeostasis, yet their dysregulated activation during neurodegeneration can sustain inflammatory cascades and worsen neuronal stress. Accordingly, strategies that jointly modulate glial activation states and inhibit pyroptotic signaling could offer dual benefits for neuroprotection [172]. In practical terms, careful tuning of microglial phenotypes together with targeted suppression of pyroptosis may help preserve neuronal integrity in disorders such as AD and PD, though the optimal combinations remain to be defined [73, 173].

Given PARP1’s central role in parthanatos, direct PARP1 inhibition has attracted interest as a complementary approach. Because parthanatos has been implicated in neuronal loss across several neurodegenerative contexts, targeting PARP1 could limit downstream injury; its involvement in NAD⁺/ATP depletion, DNA-damage responses, and AIF translocation further supports its relevance [80, 174]. At the same time, PARP1 is integral to DNA repair and cellular homeostasis, raising concerns about adverse effects with prolonged inhibition [174]. This caveat underscores the need for selective, context-aware inhibitors that balance neuroprotection with preservation of essential cellular functions, an aim highlighted in recent work [80, 174]. Representative therapeutic strategies directed at ferroptosis, necroptosis, pyroptosis, and parthanatos are summarized in Table 3, which collates findings from diverse preclinical neurodegeneration models.

Rigorous pharmacodynamic evaluation of therapeutic agents is central to clinical translation, because it verifies on-target pathway engagement, links exposure to effect, and informs dose selection and patient stratification. Integrated in silico modeling and high-throughput screening prioritize chemotypes by predicted binding and pharmacokinetics; the same pharmacodynamic benchmarks in cell systems then verify on-pathway activity and help set exposure targets ferroptosis: lower C11-BODIPY/LiperFluo oxidation and 4-HNE/MDA adducts with support of GPX4/system Xc^**−**^ and preserved mitochondrial respiration/ATP [156, 157, 175]; necroptosis: decreases in p-RIPK1/p-MLKL with DAMP/cytokine panels [58]; pyroptosis: attenuation of GSDMD-N and IL-1β/IL-18 [28]; parthanatos: reduced PAR accumulation, less AIF nuclear translocation, and partial recovery of NAD⁺/ATP [80, 174]. Clinically, relevance may be greatest in phenotypes with combined oxidative-inflammatory burden and multimodal cell-death engagement, where these panels can document on-target engagement and inform early-phase dosing. BBB penetration remains a practical bottleneck; approaches under evaluation include CNS-activated prodrugs, efflux-bypassing chemotypes, and nano/EV-based carriers to raise brain exposure while limiting off-target effects [176, 177]. Key constraints to acknowledge include, for ferroptosis, variable inducer specificity and adaptive resistance (e.g., SLC7A11/GPX4/ACSL4 dependence, transsulfuration/MT1G) alongside off-target antioxidant/chelator liabilities [178]; for necroptosis, species-dependent differences across the RIPK1-RIPK3-MLKL axis and the practical challenge of directly drugging the pseudokinase MLKL (with risks of off-target or even paradoxical effects), together with the pathway’s context-dependent immunomodulatory roles [179, 180]; for pyroptosis, selectivity host-defense trade-offs with NLRP3 blockade (dosing must avoid broad IL-1β/IL-18 suppression), translational gaps due to human caspase-4/5 vs. murine caspase-11 differences, and infection surveillance concerns when inflammasome/caspase signaling is broadly inhibited and for NLRP3 lingering safety concerns around covalent inhibitors (irreversibility/off-target reactivity) [181–183]; and for parthanatos, chronic PARP1 blockade can disrupt DNA repair (e.g., PARP-trapping), cause off-target effects on other PARPs, and be constrained by CNS exposure favoring selective, CNS-penetrant agents and careful dosing [22, 79]. Given the likelihood of long-term treatment in neurodegeneration, pharmacodynamic confirmation should be paired with longitudinal safety/tolerability monitoring and, where feasible, EV-based biomarkers, to guide progression to later-phase trials [184, 185].

**Table 3:** Summary of therapeutic compounds in preclinical models of neurodegenerative diseases

Cell death mechanisms	Related disease	Study model	Compound, pharmacological agent, drug	Therapeutic target	References
Ferroptosis	AD	In vitro	Edaravone	TLR4, lipid peroxidation	[186]
Ferroptosis	AD	In vitro	Senegenin	GPX4, ACSL4	[187]
Ferroptosis	AD	In vivo	Ginkgolide B	Nrf2/GPX4	[188]
Ferroptosis	AD	In vivo	Tetrahydroxy stilbene glycoside	Nrf2/GPX4	[189]
Ferroptosis	AD	In vivo	Deferoxamine	GSH, MDA, ROS	[82]
Ferroptosis	AD	In vivo	γ-glutamylcysteine	GSH, GPX4	[190]
Ferroptosis	AD	In vivo	α-Lipoic acid	GPx4/iron	[191]
Ferroptosis	AD	In vivo	GW7647	GPX4, iron	[192]
Ferroptosis	AD	In vivo	1,6-O, O-diacetylbritannilactone	GSH, MDA	[193]
Ferroptosis	AD	In vitro, in vivo	*Insamgobonhwan* (contents: *Liriope platyphylla*,* Asparagus cochinchinensis*,* Rehmania radix preparata*,* Ginseng radix*)	GPX4/HO-1/COX-2	[194]
Ferroptosis	AD	In vitro, in vivo	Forsythoside A	Nrf2/GPX4	[195]
Ferroptosis	AD	In vitro, in vivo	Salidroside	Nrf2/HO1/GPX4/ ACSL4, iron	[196, 197]
Ferroptosis	AD	In vitro, in vivo	Forsythoside A	Nrf2, GPX4, iron	[195]
Ferroptosis	AD	In vitro, in vivo	Eriodictyol	GPX4, Nrf2, HO-1	[198]
Ferroptosis	PD	In vitro	Idebenone	GPX4, Lipid peroxidation	[199]
Ferroptosis	PD	In vitro	α-Lipoic acid	PI3K/Akt/Nrf2, GPX4, SLC7A11	[112]
Ferroptosis	PD	In vitro	Deferoksamin	GPX4, FTH1, DMT1, TfR1	[200]
Ferroptosis	PD	In vitro	Paeoniflorin	Akt/Nrf2/Gpx4	[201]
Ferroptosis	PD	In vitro	Bafilomycin A1	FTH1, GPX4	[202]
Ferroptosis	HD	In vitro	Ferrostatin-1/ Deferoksamin	NOX2, iron-dependent lipid peroxidation	[203]
Ferroptosis	ALS	In vitro, in vivo	RTA-408	Nrf2/Gpx4, SLC7A11	[204]
Necroptosis	AD	In vitro	Necrostatin-1	RIP1	[205]
Necroptosis	AD	In vivo	Necrosulfonamide	MLKL	[206]
Necroptosis	AD	In vivo	Pazopanib	RIPK1, RIPK3, MLKL	[207]
Necroptosis	AD	In vivo	Dabrafenib - Ponatinib	pRIPK1, pRIPK3, pMLKL	[51]
Necroptosis	PD	In vivo	Necrosulfonamide	MLKL	[208]
Necroptosis	PD	In vitro, in vivo	Necrostatin-1	RIP1, RIP3, MLKL	[209, 210]
Pyroptosis	AD	In vitro, in vivo	MCC950	NLRP3	[211, 212]
Pyroptosis	PD	In vitro, in vivo	MCC950	NLRP3	[213–217]
Pyroptosis	PD	In vitro, in vivo	OLT1177	NLRP3	[218]
Parthanatos	AD	In vivo	Olaparib and MC2050	PARP-1	[219]
Parthanatos	HD	In vivo	Olaparib	PARP-1	[220]

AD: Alzheimer’s disease, PD: Parkinson’s disease, ALS: amyotrophic lateral sclerosis, HD: Huntington’s disease, TLR4: Toll-like receptor 4, GPX4: Glutathione peroxidase 4, ACSL4: Acyl-CoA synthetase long-chain family member 4, Nrf2: Nuclear factor erythroid 2–related factor 2, HO-1: Heme oxygenase 1, COX-2: Cyclooxygenase-2, GSH: Glutathione, MDA: Malondialdehyde, ROS: Reactive oxygen species, SLC7A11: Solute carrier family 7 member 11 (xCT), FTH1: Ferritin heavy chain 1, DMT1: Divalent metal transporter 1, TfR1: Transferrin receptor 1, AKT: Protein kinase B (PKB), NOX2: NADPH oxidase 2, RTA-408: Synthetic triterpenoid, Nrf2 activator, RIP1 (RIPK1): Receptor-interacting protein kinase 1, RIP3 (RIPK3): Receptor-interacting protein kinase 3, MLKL: Mixed lineage kinase domain-like pseudokinase, pRIPK1 / pRIPK3 / pMLKL: Phosphorylated forms of RIPK1, RIPK3, MLKL, NLRP3: NOD-like receptor family pyrin domain containing 3

## Integrative framework and future perspectives

Building on the pathway-level pharmacodynamic anchors and the crosstalk nodes outlined above, this framework links shared drivers (oxidative stress, lipid peroxidation, iron imbalance, mitochondrial dysfunction, ion flux, DNA damage) to cell-type programs and clinical phenotypes We propose a multi-scale framework that integrates ferroptosis, necroptosis, pyroptosis, and parthanatos into a single, action-oriented network; this approach is consistent with the inter-pathway interactions identified in recent reviews [6, 221, 222]. Oxidative stress/lipid peroxidation, iron imbalance, mitochondrial dysfunction, ion flux disorders and DNA damage/PARP1 hyperactivation stand out as common drivers of this network [178, 223]. At the level of execution modules, ferroptosis is characterized by the GPX4/SLC7A11/ACSL4 axes, necroptosis by the RIPK1-RIPK3-MLKL axes, pyroptosis by the NLRP3-caspase-4/5/11-GSDMD axes, and parthanatos by the PARP1-AIF axes [20]. A critical example of crosstalk is that MLKL-dependent K⁺ efflux facilitates the pyroptotic response by lowering the NLRP3 threshold [146, 224]. Furthermore, lipid peroxidation/mitochondrial ROS can potentiate necroptosis, highlighting the decisive role of lipid oxidation in necroptosis regulation [30]. In parallel, excessive PARP1 activation deepens mitochondrial failure and creates conditions that may increase ferroptotic sensitivity [225, 226]. Neurons are particularly prone to ferroptosis due to their iron-rich lipidome and energy fragility [227]. In microglia, the NLRP3 inflammasome distinctly shapes pyroptotic threshold and neuroinflammation [172]. Astrocytes contribute to the maintenance of GPX4 activity by regulating glutathione/selenium metabolism, thereby gatering ferroptosis susceptibility [228, 229]. Triggers such as amyloid or α-synuclein accumulation, excitotoxicity, and environmental toxins engage this network through different entry points, leading to convergent outcomes such as synaptic loss, axonal degeneration, and maladaptive neuroinflammation [77, 230–233].

For translational roadmap efforts, we recommend a composite biomarker approach that maps a panel of pathway-proximal reads to EV-based signatures [234]. Recent studies that activation of non-canonical regulated cell death programs generates pathway-proximal markers that are actively packaged into extracellular vesicles (EVs) via endo-lysosomal remodeling [234–236]. These EV-encapsulated markers can cross the blood-brain barrier and provide an accessible window into neurodegenerative processes measurable in plasma or cerebrospinal fluid. Proof-of-concept studies have documented p-MLKL-positive EVs released during necroptosis, GSDMD-N bearing EVs arising from pyroptotic cells, and 4-HNE–modified proteins within circulating EVs after oxidative injury [57, 237–239]. Complementing these mechanistic insights, a recent meta-analysis suggests that points to the potential diagnostic utility of EV-based assays [240]. Incorporating pathway-proximal cell-death biomarkers into these panels could enable clinicians to (i) stratify patients by the dominant death pathway involved, (ii) match them with pathway-targeted interventions, and (iii) monitor on-target drug engagement in near real time capabilities often cited as prerequisites for precision medicine. Accordingly, recent reviews tend to frame EV-derived biomarkers as a promising bridge between molecular diagnosis and patient-tailored therapy in neurodegeneration.

On the therapeutic front, several complementary or sequential approaches could be explored: (i) mitigating ferroptosis with radical-trapping antioxidants such as ferrostatin-1 or liproxstatin-1, together with selenium-GPX4 support and programmes that up-regulate SLC7A11; (ii) tempering necroptotic escalation via RIPK1 inhibitors; (iii) easing pyroptosis through NLRP3 modulators (e.g., MCC950); and (iv) restoring metabolic resilience with context-specific PARP inhibitors (e.g., Deferiprone) [172, 209, 241–244]. The therapeutic compounds summarised in Table 3 each mapped to disease-specific biomarkers provide further support for this integrated intervention framework. Moving forward, it would be helpful to: (i) gather clinical evidence to support composite biomarker panels, (ii) refine the dosing and sequencing principles for combination therapies, and (iii) promote consistent reporting of study outcomes. By harmonising molecular read-outs with cell-type context and patient presentation, this integrative framework may gently guide the progression from early exploratory studies to thoughtfully crafted clinical trial designs [221, 240].

## Conclusion

Non-canonical cell death pathways such as ferroptosis, necroptosis, pyroptosis, and parthanatos are increasingly recognized as important elements in the complex landscape of neurodegenerative diseases. Current evidence suggests that these processes may act both as contributors to neuronal injury and as potential therapeutic entry points. While encouraging observations have been made in preclinical models, the extent to which this transpires in clinical benefit is yet to be determinedIn this regard, the incorporation of cell death signatures into biomarker-guided methods, could be useful for patient stratification into more appropriately defined therapy decisions. Multi-target strategies employing simultaneous means of addressing oxidative stress, inflammation and metabolic dysregulation, may provide a wider benefit but must be further evaluated for safety and long-term efficacy. Improvements in drug delivery systems that can cross the blood-brain barrier and developments in omics-based translational models may increase avenues for success in clinical practice. In addition, knowing the connections between regulated forms of cell death may promote combination therapies among these pathways, but this also complicates clinical strategies.

Taken together, while these emerging insights are promising, further efforts are needed to validate mechanistic findings in human studies and to develop safe, precise, and durable interventions. A systems level perspective that integrates molecular mechanisms with clinical context may eventually contribute to the development of disease-modifying strategies for neurodegenerative disorders.

## Data Availability

No datasets were generated or analysed during the current study.
