# Correction: Non-canonical cell death in neurodegeneration: emerging mechanisms and therapeutic Frontiers

**DOI:** 10.1007/s10495-026-02387-y

**Published:** 2026-07-04

**Authors:** Nilufer Ercin, Nail Besli, Merve Beker, Ulkan Celik

**Affiliations:** 1 Department of Medical Biology, Hamidiye School of Medicine, University of Health Sciences, Istanbul, Turkey; 2 Department of Medical Biology, Hamidiye International School of Medicine, University of Health Sciences, Istanbul, Turkey; 3 Department of Medical Biology, Institute of Health Sciences, University of Health Sciences, Istanbul, Turkey

**Correction to: Apoptosis (2026) 31:72** 10.1007/s10495-026-02260-y

In this article, the caption for Fig. 3 was inadvertently placed in page 11 as Eigth paragraph. The figure should have appeared as shown below.

**Fig. 3:**
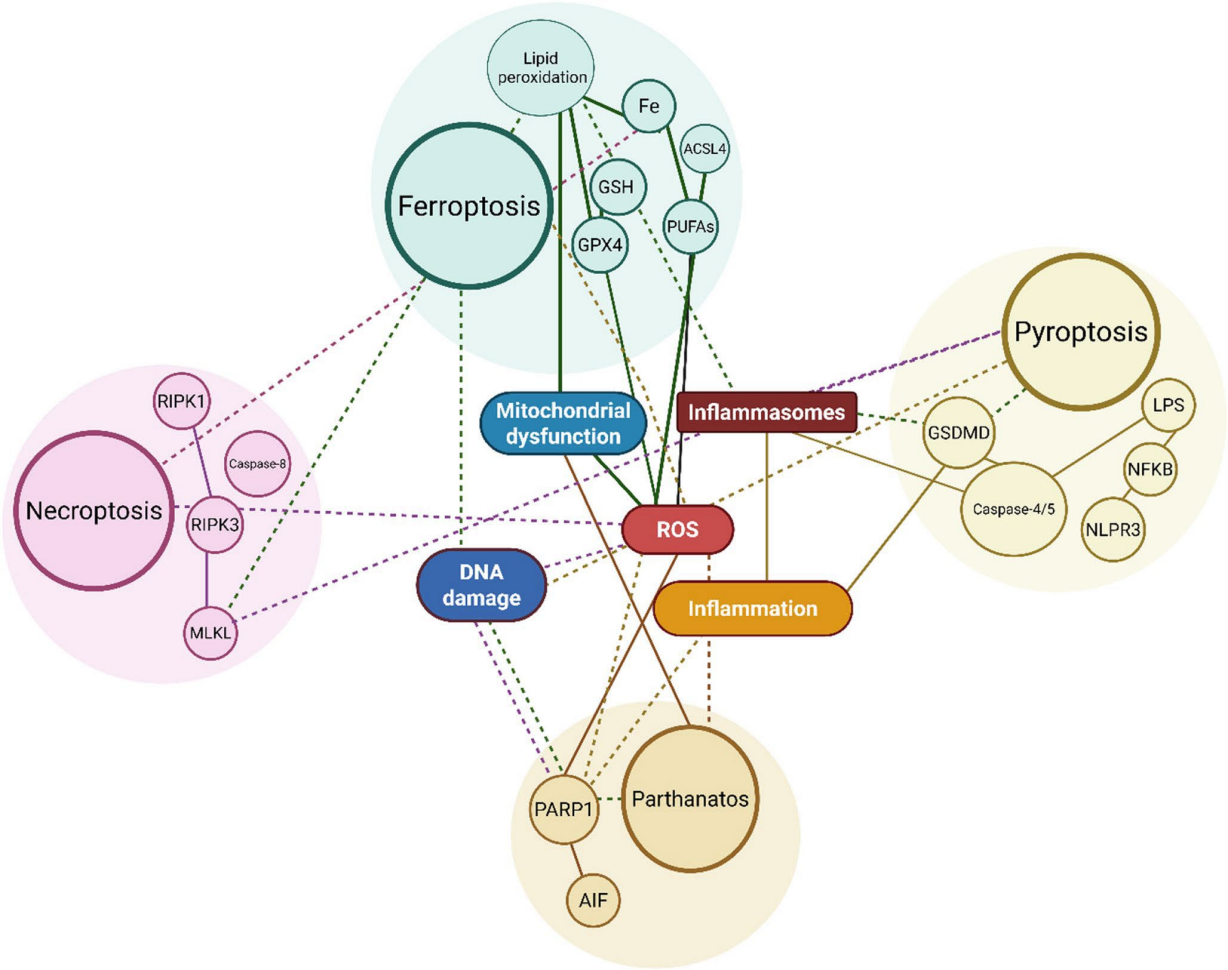
Interaction network among non-canonical cell death pathways. This schematic diagram illustrates the molecular interactions of ferroptosis, necroptosis, pyroptosis, and parthanatos, along with their connections to shared pathological processes. Solid lines represent direct molecular interactions and biochemical relationships. Dashed lines indicate indirect associations or the influence of one pathway on another. Created in https://BioRender.com/1bv771o

In addition, footnote for Table 3 was inadvertently placed in page 13 as Sixth paragraph. The Table should have appeared as shown below.

**Table 3:** Summary of therapeutic compounds in preclinical models of neurodegenerative diseases

Cell death mechanisms	Related disease	Study model	Compound, pharmacological agent, drug	Therapeutic target	References
Ferroptosis	AD	In vitro	Edaravone	TLR4, lipid peroxidation	[186]
Ferroptosis	AD	In vitro	Senegenin	GPX4, ACSL4	[187]
Ferroptosis	AD	In vivo	Ginkgolide B	Nrf2/GPX4	[188]
Ferroptosis	AD	In vivo	Tetrahydroxy stilbene glycoside	Nrf2/GPX4	[189]
Ferroptosis	AD	In vivo	Deferoxamine	GSH, MDA, ROS	[82]
Ferroptosis	AD	In vivo	γ-glutamylcysteine	GSH, GPX4	[190]
Ferroptosis	AD	In vivo	α-Lipoic acid	GPx4/iron	[191]
Ferroptosis	AD	In vivo	GW7647	GPX4, iron	[192]
Ferroptosis	AD	In vivo	1,6-O, O-diacetylbritannilactone	GSH, MDA	[193]
Ferroptosis	AD	In vitro, in vivo	*Insamgobonhwan* (contents: *Liriope platyphylla*, *Asparagus cochinchinensis*, *Rehmania radix preparata*, *Ginseng radix*)	GPX4/HO-1/COX-2	[194]
Ferroptosis	AD	In vitro, in vivo	Forsythoside A	Nrf2/GPX4	[195]
Ferroptosis	AD	In vitro, in vivo	Salidroside	Nrf2/HO1/GPX4/ ACSL4, iron	[196, 197]
Ferroptosis	AD	In vitro, in vivo	Forsythoside A	Nrf2, GPX4, iron	[195]
Ferroptosis	AD	In vitro, in vivo	Eriodictyol	GPX4, Nrf2, HO-1	[198]
Ferroptosis	PD	In vitro	Idebenone	GPX4, Lipid peroxidation	[199]
Ferroptosis	PD	In vitro	α-Lipoic acid	PI3K/Akt/Nrf2, GPX4, SLC7A11	[112]
Ferroptosis	PD	In vitro	Deferoksamin	GPX4, FTH1, DMT1, TfR1	[200]
Ferroptosis	PD	In vitro	Paeoniflorin	Akt/Nrf2/Gpx4	[201]
Ferroptosis	PD	In vitro	Bafilomycin A1	FTH1, GPX4	[202]
Ferroptosis	HD	In vitro	Ferrostatin-1/ Deferoksamin	NOX2, iron-dependent lipid peroxidation	[203]
Ferroptosis	ALS	In vitro, in vivo	RTA-408	Nrf2/Gpx4, SLC7A11	[204]
Necroptosis	AD	In vitro	Necrostatin-1	RIP1	[205]
Necroptosis	AD	In vivo	Necrosulfonamide	MLKL	[206]
Necroptosis	AD	In vivo	Pazopanib	RIPK1, RIPK3, MLKL	[207]
Necroptosis	AD	In vivo	Dabrafenib - Ponatinib	pRIPK1, pRIPK3, pMLKL	[51]
Necroptosis	PD	In vivo	Necrosulfonamide	MLKL	[208]
Necroptosis	PD	In vitro, in vivo	Necrostatin-1	RIP1, RIP3, MLKL	[209, 210]
Pyroptosis	AD	In vitro, in vivo	MCC950	NLRP3	[211, 212]
Pyroptosis	PD	In vitro, in vivo	MCC950	NLRP3	[213–217]
Pyroptosis	PD	In vitro, in vivo	OLT1177	NLRP3	[218]
Parthanatos	AD	In vivo	Olaparib and MC2050	PARP-1	[219]
Parthanatos	HD	In vivo	Olaparib	PARP-1	[220]

AD: Alzheimer’s disease, PD: Parkinson’s disease, ALS: amyotrophic lateral sclerosis, HD: Huntington’s disease, TLR4: Toll-like receptor 4, GPX4: Glutathione peroxidase 4, ACSL4: Acyl-CoA synthetase long-chain family member 4, Nrf2: Nuclear factor erythroid 2–related factor 2, HO-1: Heme oxygenase 1, COX-2: Cyclooxygenase-2, GSH: Glutathione, MDA: Malondialdehyde, ROS: Reactive oxygen species, SLC7A11: Solute carrier family 7 member 11 (xCT), FTH1: Ferritin heavy chain 1, DMT1: Divalent metal transporter 1, TfR1: Transferrin receptor 1, AKT: Protein kinase B (PKB), NOX2: NADPH oxidase 2, RTA-408: Synthetic triterpenoid, Nrf2 activator, RIP1 (RIPK1): Receptor-interacting protein kinase 1, RIP3 (RIPK3): Receptor-interacting protein kinase 3, MLKL: Mixed lineage kinase domain-like pseudokinase, pRIPK1 / pRIPK3 / pMLKL: Phosphorylated forms of RIPK1, RIPK3, MLKL, NLRP3: NOD-like receptor family pyrin domain containing 3

The original article has been corrected.

